# Book Reviews

**Published:** 1894-04

**Authors:** 


### Book Reviews.

AN ILLUSTRATED ENCYCLOPEDIC MEDICAL DICTIONARY. Being a dictionary of technical terms used by writers on medicine and the collateral sciences, in the Latin, English, French and German languages. By FRANK P. FOSTER, M. D., editor of the *New York Medical Journal*, assisted by eleven collaborators. Vol. IV. Minn-Zyth. With illustrations. Quarto, pp. 776. D. Appleton & Co. 1894.

The completion of this colossal enterprise marks an epoch in wordbook making. It becomes a fit occasion on which to congratulate the editor and publishers, as well as all who have had to do with the construction of this great work. Volume IV. is in all respects a parallel to its predecessors in form, style and general makeup, which in detail means that it is printed on heavy book-paper, in double columns with nonpareil caps for words and the definitions are given in plain nonpareil with compounds in capitalized full-faced nonpareil type.

It is copiously illustrated with fine engravings wherever essential to the better understanding of the text. This dictionary has no rival, because it is the only one extant in medical literature that is of a strictly encyclopedic character. The aggregate number of pages in the four volumes is 3,096, and when it is remembered that these are double-column, quarto, closely printed pages, it will be readily appreciated that an enormous amount of information is compressed into each volume separately, as well as into the work as a whole.

While, as we have remarked heretofore, the pages are closely printed, there has yet been such an admirable selection of type as to preclude eye strain in their reading. As a frontispiece to the fourth volume there is an excellent plate on which is engraved twenty-three figures, in colors, showing the principal normal and abnormal constituents of human urine, compiled and redrawn from Peyer's atlas of clinical microscopy and other sources. This plate is accompanied by a complete reference table, making it a most valuable aid to the histological study of the urine.

We have remarked in notices of the separate volumes, that this is a dictionary that every library ought to possess, whether medical or general, and one that every teacher and practitioner who would do himself justice should obtain. We desire to accentuate this opinion as we close this final notice of the most successful and satisfactory wordbook that has ever been uttered in English or any other tongue. The work is so strongly and substantially constructed and bound, that it will last many years even under the most constant use.

---

Annual Report of the State Board of Charities for the year 1892, Transmitted to the Legislature January 26, 1893. Octavo, pp. 591. Albany: James B. Lyons, State Printer. 1893.

The reports of this board always possess great interest to philanthropists as well as all public-spirited citizens. In it are discussed questions pertaining to the poor, the insane, epileptics, idiotic and feeble-minded, the blind, the deaf, the criminal, the orphan and the veteran. This board is doing an excellent work in sending alien paupers to their homes in different countries in Europe. During the year covered by this report, 150 such, who had been deported to this country from their several European homes were returned. Of this number there were lunatics, 9; imbeciles, 6; epileptics, 3; paralytics, 5; vagrants and diseased, 27; old and decrepit, 22; blind, 2; crippled, 7; deformed, 4; feeble-minded, 26; otherwise diseased, 39; total, 150. Could any more potent argument be offered for the supervision of immigration and the strictest enforcement of laws relating thereto?

In this report are given the details of the selection of a site for and the organization of an epileptic colony. The property formerly owned by the Shakers at Sonyea, Livingston County, was chosen and is admirably adapted to the purposes named. The buildings are fully illustrated in the volume and a map of the property accompanies it.

---

An American Text-Book of Gynecology, Medical and Surgical. For the Use of Students and Practitioners. By Henry T. Byford, M. D., John M. Baldy, M. D., Edwin Cragin, M. D., J. H. Etheridge, M. D., William Goodell, M. D., Howard A. Kelly, M. D., Florian Krug, M. D., E. E. Montgomery, M. D., William R. Pryor, M. D., George M. Tuttle, M. D. Edited by J. M. Baldy, M. D. Forming a handsome royal octavo volume, with 360 illustrations in text, and thirty-seven colored and half-tone plates. Price, cloth, $6.00; sheep, $7.00; half Russia, $8.00. Pp. xxiv.—713. For sale by subscription. Philadelphia: W. B. Saunders, 925 Walnut street. 1894.

Treatises on gynecology are following each other in such rapid succession that it almost takes one's breath away, metaphorically speaking, to keep up with the rapid pace set by authors and publishers. That there is some excuse for the appearance of these works with a reasonable degree of rapidity, speaking with reference to the intervals of publication, cannot be denied. The alleged necessity for these rapid strides in gynecological book-making may be accounted for in part by the advances in the science and art in gynecology, and in other part by the ambition of physicians to pose as authors, and in still other part by the desire of publishers to carry on their work and to make it as profitable as possible. Nevertheless, each work possesses some advantages over its predecessors, to which rule the present treatise is no exception.

The opening section of the book is devoted to a consideration of the examination of the female pelvic organs. The usual directions are given with reference to the office examination, in the course of which an examination table and chair, together with a gynecological cabinet, are illustrated. We are at a loss to understand why any form of so-called gynecological chair should receive even the *quasi* indorsement of pictorial display in a modern gynecological treatise. A moderate space is given to the consideration of posture that we think might properly be extended with considerable advantage. An accurate knowledge of posture, not only with reference to treatment, but also as it relates to the causation of disesae, should be acquired by every physician who attempts the practice of gynecology.

In the next section of the work the technique of gynecological operations is considered. It is here properly stated that technique is the animating principle of successful operations, but that it has nothing to do with dexterity or rapidity. Sepsis, asepsis and antisepsis are briefly considered, and the operating room fully described and illustrated. The preparation of the operator and his assistants receives due attention, while ligatures, suture materials, dressings, sponges and instruments are considered with reference to sterilization and application. The description of the technique of the abdominal incision, including its opening, its closure and after-management, would have been very considerably extended with profit to students and practitioners.

Menstruation and its anomalies are considered in the next forty-two pages, and these contain most interesting and instructive reading. These questions should be more carefully studied by physicians and information thereon be more accurately imparted to mothers, and through them to daughters, than is done even in the intelligent sunlight of the present age. Sterility is briefly considered without, however, throwing any new light upon the subject, and then anomalies of the female genital organs are dealt with. Then follows genital tuberculosis, which forms an interesting study in the new light of tubercular pathology, and then diseases of the vulva and vagina are considered. This is one of the most interesting sections in the book, and is a field that deserves to be more fully explored than has heretofore been done. Almost every one of the subheads in this section could be made to serve as the proper title for an extended essay. We next come to inflammatory diseases of the uterus, that is considered in a section comprising forty-two pages. It contains numerous microscopical illustrations throwing light on the pathology of these affections and many others relating to instrumentation and treatment.

Plastic surgery of the genital tract is described in the next two sections under lacerations of the soft parts and genital fistulæ. The directions given for these operations are for the most part clear and the illustrations are generally correct; especially is this the case with reference to those pertaining to laceration of the cervix. Distortions and malpositions of the uterus are considered in a section comprising eighty pages, that furnish much interesting reading. There is room for difference of opinion as to the part played in symptomatology by some of the malpositions and distortions of the uterus, but it is well to have methods of replacement clearly defined so that intelligent application thereof may be made whenever a clear relationship of cause and effect is diagnosticated. We doubt the propriety, however, of undertaking the reposition of the uterus by any mechanical repositor, hence we take exception to the instrument on page 298, the Sims-Pryor repositor, as well as to the description of its use. The question of pessaries is always an interesting one, and permits of much latitude in opinion. The pessaries that really do good are few and the men who are competent to use them discreetly are fewer. Within certain boundaries they do good, but they are also capable of much harm. The personal equation has here a strong and solid foundation. We wish authors would discontinue illustrating all pessaries which fasten to belts outside the body. They mislead the novice and are rarely capable of good in the hands of the expert.

In the section on malignant diseases of the female genitalia will be found complete directions for the performance of vaginal hysterectomy as well as a group of well-drawn illustrations, clearly explaining much of the text. In this section the term malignant is applied to those affections which progress toward a fatal termination and have a tendency to return after removal—a most lucid and satisfactory definition. It stands out in bold relief to the subject treated of in the next section,—namely, uterine neoplasms, which are benign in character and do not tend to return after removal. In this section abdominal hysterectomy is dealt with and amply illustrated. The several methods of treating the pedicle are fully set forth, and the indorsement of the author is given to the intra-abdominal method.

Pelvic inflammation is always an interesting subject to general practitioner as well as gynecologist, and the section devoted to it is full of information and well written. Pyosalpinx, ovarian abscess and pelvic peritonitis are here fully treated, but there is still another section devoted to diseases of the ovaries and tubes in which cystoma, myoma and the like are considered. Between these two sections ectopic gestation is interpolated and all its various phenomena detailed and illustrated.

A most instructive and important section is that devoted to diseases of the urethra, bladder and ureters. Then comes the final section devoted to after-treatment in gynecological operations. This contains many well-considered directions, and in general may be said to furnish a safe guide in the post-operative conduct of gynecological cases.

Finally, it may be remarked that this work will find a useful place alongside of the other treatises that have lately been issued on subjects to which it is akin. The editor has performed a difficult task with much skill and a credit that will give him fame during the coming years.

---

Foreign Bodies in the Larynx and Trachea and in the Pharynx and Esophagus. By John O. Roe, M. D., Rochester, N. Y., Fellow of the American Laryngological Association; Corresponding Members of the Sociètè Francaise d'Otologie, de Laryngologie et de Rhinologie; Member of the British Medical Association, of the American Climatological Association, of the American Medical Association, of the Medical Society of the State of New York, of the Central New York Medical Association, of the Monroe County Medical Society, etc. Large octavo, pp. 73. Reprinted from Volume XI. of The System of Diseases of the Ear, Nose and Throat, edited by Charles H. Burnett, M. D., and published by J. B. Lippincott Company, Philadelphia. 1893.

The subjects dealt with in this monograph possess unusual interest. There are few persons who have not been annoyed at some time during their lives with at least a fish bone in their throats, the memory of which, to say the least, is not pleasant. The irritation from even so slight a body as a small fish bone is often so great as to cause alarming anxiety. The variety of foreign bodies that occasionally lodge in the larynx and trachea and in the pharynx and esophagus is very great, though by far the most common objects, according to Roe, are fruit stones, pebbles, grains of corn, beans, coins and buttons. Among the curious bodies that have been occasionally found in these passages are puff-darts, a toy velocipede, brass boot hook, a large brooch, shawl pin, an iron buckle, an amber cigar holder, a toy balloon and the mouth-piece of a trumpet. Roe gives numerous illustrations of a number of foreign bodies *in situ*, among which may be mentioned a toy locomotive in the larynx, piece of boiled beef, a buckle, a dime, brass watch ring and a cockle burr. Other illustrations are given of foreign bodies in the esophagus, one of the most common of which is a tooth plate.

All the various methods of extraction are given in this brochure, and instruments used for that purpose are extensively illustrated. Roe has grouped in a single monograph a vast amount of information which renders it unnecessary to search the literature through and through to obtain intelligence concerning any given case, for here they are noted with complete references. It is one of the most satisfactory contributions to the literature of the subject that has ever been made, and no one could have been chosen for this work who is better equipped than this author.

---

Chemistry and Physics. By Joseph Struthers, Ph. B., Columbia College School of Mines, New York ; D. W. Ward, Ph. B., Columbia College School of Mines, New York ; and Charles H. Willmarth, M. S., New York. $1.00. The Students' Quiz Series. Philadelphia : Lea Brothers & Co. 1893.

This number of the Students' Quiz Series has more place and a larger excuse for its existence than most of such condensations of medical literature. To the majority of students the subject of chemistry is the *bête noir* of medical study, hence any method of imparting such knowledge and fixing it in the memory, even though it goes only part way toward the accomplishment of the object, is to be commended. Students will find this compend of much use in the pursuit of their studies.

---

Transactions of the American Gynecological Society. Volume VIII., for the year 1893. Small 8vo, pp. xl.—542. Philadelphia : William J. Dornan, Printer. 1893.

This interesting volume appears with promptitude, as usual, and contains an excellent group of papers, many of which are discussed in a pertinent and strong manner. The president's address, by Dr. Theophilus Parvin, attracts attention from two points of view. First, he paid a graceful compliment to the American Association of Obstetricians and Gynecologists, admitting it to be a formidable rival national association. He said:

"That association numbers many able members, and has done very creditable and useful work." Regarding the proposition of the amalgamation of the two societies, he recorded himself as decidedly against such a project, for, he continued, the country is too large, the number of the profession too great for the amalgamation of the two organizations. Moreover, he spoke in decided opposition to a duality of membership, because, he affirmed, proselyting is neither pleasant nor promising, and there is work enough for each organization. His words on this subject deserve to be pondered by every member of both societies, and we here reproduce them in part, as follows:

> Nor do I believe that professional polygamy should be encouraged; monogamy ought to be the rule, and even bigamy a rare exception. Possibly doctors may sometimes want double honors, or triple, as a bashaw is not content with one tail, but seeks two or three as symbols of his power and importance. A doctor has at times been called to a young child suffering with digestive disorder, and to his inquiry as to its diet, he is told: Oh, it sits at the table and takes everything that is going.
>
> A divided is still too often a doubtful allegiance, and I believe that a man ought to be satisfied to be a member of either organization. Moreover, the American Gynecological Society—good members as it has received from its rival, good men as it may now or in the future have the opportunity of receiving—has not room for the reception of such applicants without excluding equally qualified men who do not belong to any similar organization.

After this eloquent and just rebuke, it is to be hoped that no further attempts will be made on the part of the older society to subtract from the younger any of its members.

The second point in this address to which we would call attention, is Dr. Parvin's advocacy of a reamalgamation of the chairs of obstetrics and gynecology in the medical schools. Impressed with the importance of this subject, he wrote to Professor Winckel asking the reasons for the practice, universally prevalent in Germany, of uniting obstetrics and diseases of women under one teacher. Winckel's communication follows the president's address, and is an elaborate setting forth of this view of the subject. It is in such complete juxtaposition to American ideas and practice that we commend it to the careful study of all interested. There is one brilliant example in this country where a union of the practice of the two departments in a single individual has given results of a most resplendant character. Dr. Joseph Price, of Philadelphia, who is concededly the foremost American abdominal surgeon, has, as physician in charge of the Preston Retreat during the past six years, demonstrated his competency as an obstetrician, by presenting to the profession the most striking and satisfactory record made by any maternity physician in the world—namely, more than thirteen hundred consecutive confinements, including operative cases of the most serious character, without a death.

As we said at the outset, this book is full of interesting reading, and will well repay a careful perusal.

---

A Manual of Diseases of the Nervous System. By W. R. Gowers, M. D., F. R. C. P., F. R. S., Consulting Physician to University College Hospital; Physician to the National Hospital for the Paralyzed and Epileptic. Second edition, revised and enlarged. Volume II. Diseases of the Brain and Cranial Nerves, General and Functional Diseases of the Nervous System. With 182 illustrations, including a large number of figures. Octavo, pp. xvi.—1069. Philadelphia: P. Blakiston, Son & Co., 1012 Walnut street. 1893.

The reputation of the author is such that a review of his work is in reality a review of our present knowledge of nervous disease. Whatever Professor Gowers treats he does so fully and from his own experience. This volume, as its title indicates, considers the diseases of the brain, cranial nerves and the functional diseases. The first sixty pages deal purely with the structure and function of the brain, considering in detail the cerebral cortex, its relation to the skull and its functional regions of sight, speech, hearing, smell and centers of motion. This is most clearly done and elucidated by means of reports of cases, illustrated by nineteen plates.

The connecting tracts and ganglias, the cranial nerves and their origins are next considered, and the subject matter is rendered easy of understanding by numerous illustrations.

In his discussion of the cerebellum, it is profitable to note that in these days of exact localization of brain function, he is obliged to say (page 59): "The function of the cerebellum is still mysterious." "The simple loss of substance causes no definite and recognizable loss of any function of the brain."

"The loss can apparently be compensated by other parts. Hence it is possible that the old theory may be correct, that the cerebellar hemispheres are in some way connected with psychical processes."

In his study of the cranial nerves and the clinical symptoms to which each may give rise, one is impressed with the thoroughness of the work done, especially in his consideration of the ocular nerves, which is more complete in detail than in any other work on the subject.

The book everywhere shows a careful revision of the first edition. In his classification of causes of paralysis of the ocular muscles, in addition to eight causes given in edition one, he mentions hemorrhage into the sheath of a nerve in Goldscheider's case in 1892. This is mentioned as an example of the minute care in this edition to bring it up to the latest knowledge.

In the article on meningitis, he mentions (page 328) serous pachymeningitis, which is not referred to in edition one.

The article on actinomycosis and also the one on astasia abasia is entirely new, no reference being made to them in edition one.

The recent advances in cerebral surgery have led the author to advocate surgical measures in sinus thrombosis, which he did not do previously. He refers to Ballance, who opened the sinus after double ligature of the jugular vein and then cleared out the thrombus with satisfactory results, and without extension to the opposite sinus.

Of acute cerebral palsies of children, he says that valuable analyses of cases have been published since the first edition of this work, and refers to Osler-Sachs and to Volkmann's Vorträge, 1892, Die Hirnlahmunger der Kinder.

In his first edition the subject of tetanus was fully up to the date of its writing. In the second edition the bacillus is mentioned, and the entire theory of the disease is changed.

In his treatment of diseases we notice that the author has accepted the new drugs antipyrin, acetanilide, exalgin and phenacetin. In his first edition antipyrin was not recommended.

Concerning these drugs in his treatment of neuralgia, he says: "In some cases, marked relief is afforded by them ; they are somewhat uncertain in their influence, which is sometimes great at first and afterward slight."

In his chapter on paralysis after acute diseases, he refers to typhoid fever, typhus fever, erysipelas, variola, measles, scarlet fever, mumps, malarial fever, dysentery, simple diarrhea, acute rheumatism, influenza and diphtheria. Of these, influenza as a cause of paralysis was not mentioned in edition one. This is interesting, because edition one was written in 1888. He says there is no acute malady with the exception of diptheria, after which the disturbance of the nervous system is so frequent as after influenza. This effect has never been perceived so distinctly as in the last four years, 1890-93.

Under the varieties of nervous disturbances we find definite melancholia, mental dulness, mania hysteria, convulsions of epileptic character, intercostal neuritis, multiple neuritis, meningitis, hemorrhages, cerebritis and myelitis. Thus we see that the sequelæ of influenza are extremely varied, and that they are far more to be dreaded than the disease itself.

That the author is an Englishman accounts, perhaps, for the fact that he does not mention the fact of eye strain in headaches, migraine, epilepsy, or chorea. This is not because he has not studied the subject, but evidently it is because he has not found that eye strain is a cause of epilepsy, or chorea of headache, or a migraine. We are not surprised at the fact that he does not mention ocular errors in connection with the first named diseases, but we would like to have had a definite opinion from him on the relation of headache to eye strain.

In this book we also look in vain for articles on myxedema and acromegaly. Perhaps he does not consider them as nervous in origin, and therefore should have no place in a treatise strictly on diseases of the nervous system. They are, however, treated in the American books on nervous diseases.

Taken as a whole, the work of Prof. Gowers is a most comprehensive and judicial one. His opinion on doubtful points is always given and with it his reasons for it.

The treatment is perhaps the least satisfactory, but perhaps that is because of his extreme honesty. No better book on the subject has appeared in any language, and as we have shown it is thoroughly revised up to date.

The illustrations are numerous, but in many instances are poor. The type and paper are good. J. W. P.

---

AN OUTLINE OF THE EMBRYOLOGY OF THE EYE, with illustrations from original pen-drawings by the author. By WARD A. HOLDEN, A. M., M. D., assistant surgeon New York Ophthalmic and Aural Institute, Clinical Assistant Vanderbilt Clinic. The Cartwright Prize Essay for 1893, pp. 69, 12mo. G. P. Putnam's Sons, New York and London. 1893.

This is a clear and concise exposition of the embryological development of the eye. The author has studied the subject experimentally, and his excellent descriptions are illustrated by a connected series of equally excellent engravings, mostly from drawings by himself. The work is to be highly commended.

A. A. H.

---

International Clinics. A Quarterly of Clinical Lectures on Medicine, Neurology, Pediatrics, Surgery, Genito-Urinary Surgery, Gynecology, Ophthalmology, Laryngology, Otology and Dermatology. By professors and lecturers in the leading medical colleges of the United States, Great Britain and Canada. Edited by John M. Keating, M. D., LL. D., Colorado Springs, Col.; Fellow of College of Physicians, Philadelphia; formerly Consulting Physician for Diseases of Women to St. Agnes' Hospital; Gynecologist to St. Joseph's Hospital; Visiting Obstetrician to the Philadelphia Hospital, and Lecturer on Diseases of Women and Children, Philadelphia; Editor Cyclopedia of the Diseases of Children. Judson Daland, M. D., Philadelphia, Instructor in Clinical Medicine, and Lecturer on Physical Diagnosis and Symptomatology, in the University of Pennsylvania; Assistant Physician to the University Hospital; Physician to the Philadelphia Hospital and to the Rush Hospital for Consumption. J. Mitchell Bruce, M. D., F. R. C. P., London, England, Physician and Lecturer on Therapeutics at the Charing Cross Hospital. David W. Finlay, M. D., F. R. C. P., Aberdeen, Scotland, Professor of Practice of Medicine in the University of Aberdeen; Physician to, and Lecturer on, Clinical Medicine in the Aberdeen Royal Infirmary; Consulting Physician to the Royal Hospital for Diseases of the Chest, London. Volume II. Third series, 1893. Royal octavo, pp. xii.—363. Philadelphia: J. B. Lippincott Co. 1893.

In this volume is continued the valuable series of clinical lectures that have been issued by the Lippincott Company during several years past. This volume is no exception to the rule in interesting and instructive clinical teaching. Buffalo, too, is well represented in this volume. Dr. Charles Cary presents a lecture on Gonorrheal Iritis and another on Impetigo Contagiosum; Dr. M. D. Mann one on Retained Menstrual Blood; Dr. Roswell Park one on Trephining for the Psychopathic Equivalent of Epilepsy—Craniotomy for Idiocy; there is one by Dr. W. C. Phelps on Stenosis of the Pharynx; one by Dr. James Wright Putnam on Hysteria in the Male; and one by Dr. Charles G. Stockton on Hepatic Cirrhosis. Other lectures of interest are delivered by Dr. E. E. Montgomery, of Philadelphia, Dr. Emory Lanphear, of Kansas City, Dr. Stephen Smith, of New York, Dr. E. Fletcher Ingals, of Chicago, Dr. John B. Hamilton, of Chicago, as well as many others whom we have not space to mention. It is a satisfactory companion to the numbers that have preceded it.

A Text-Book of the Theory and Practice of Medicine. By American teachers. Edited by William Pepper, M. D., LL. D., Provost and Professor of the Theory and Practice of Medicine and of Clinical Medicine in the University of Pennsylvania. In two volumes; illustrated. Vol. II. Large octavo, pp. xii.—1046. Philadelphia: W. B. Saunders, 913 Walnut street. 1894. Price per volume, cloth, $5.00; leather, $6.00; half Russia, $7.00. For sale by subscription only.

This volume contains contributions from Dr. W. H. Welch, Dr. Henry M. Lyman, Dr. William Osler, Dr. William Pepper, Dr. James C. Wilson, Dr. Francis Delafield, Dr. James W. Holland and Dr. Reginald H. Fitz.

The first article by Dr. Welch upon the biology of bacteria, infection and immunity, is one of very great value, and presents in a concise manner a close, thorough and elaborate study of these subjects. This article alone makes the volume desirable. The biology of bacteria is treated under the headings morphology and classification; food, vital manifestations — distribution; agencies injurious to bacteria; modifications of characters; attention of virulence; marks of differentiation. The data of these subjects are given up to February, 1894.

Dr. Lyman has prepared a series of articles, among which those upon saccharine diabetes and acute articular rheumatism deserve special mention. Dr. Osler's contribution upon diseases of the blood is very complete. We know of nothing that more forcibly illustrates the change that has been wrought in our methods of medical diagnosis during the past few years than does this masterly article by Dr. Osler, in which the latest devices for the examination of the blood are illustrated. Dr. Osler attaches considerable importance to the Blitz-hedin hematokrit, and thinks it will be more generally adopted as soon as a few suggested improvements have been made in it. Any instrument of precision that will furnish the busy clinician with scientific data for diagnosis is always welcome and valuable.

Dr. Pepper's prolific pen gives to this volume a large number of good contributions. Wide experience and knowledge of medicine pervade whatever Dr. Pepper writes, and we are especially fortunate in being favored by the busy editor of the volume.

Dr. Wilson contributes the sections upon diseases of the nose, larynx, bronchi and pleura. Dr. Delafield has written the chapter upon diseases of the lungs and kidneys; while good articles upon diseases of the peritoneum, liver and pancreas are from the pen of Dr. Fitz.

This volume, as a whole, is a good book for students as well as for practitioners. A. A. J.

---

Manual of Physical Diagnosis, for the Use of Students and Physicians. By James Tyson, M. D., Professor of Clinical Medicine in the University of Pennsylvania, and Physician to the University Hospital; Physician to the Rush Hospital for Consumption and Allied Diseases; Fellow of the College of Physicians of Philadelphia; Member of the Association of American Physicians, etc. Second edition, revised and enlarged. Duodecimo, pp. 241. Philadelphia: P. Blakiston, Son & Co., 1012 Walnut street. 1893. Price, $1.50.

The appearance of a second edition of this manual so soon after the issuance of the first bespeaks not only the popularity of the book but the importance of the subject, an appreciation of which is gradually growing wider and wider as medical science advances. We can only reiterate what we have said heretofore, that it is of paramount importance for the student of medicine to be well drilled in physical diagnosis, in order that he may properly interpret the phenomena of disease, and intelligently apply methods of treatment. Tyson's work is concise, instructive and valuable, and deserves to be carefully studied both by medical students and physicians.

---

Transactions of the Medical and Chirurgical Faculty of the State of Maryland. Ninety-fourth annual session, held at Baltimore, Maryland, April, 1892; also semi-annual session, held at Rockville, Md., November, 1891. Paper octavo, pp. 124. Baltimore: Griffin, Curley & Co., Printers, 202 E. Baltimore street. 1892.

Transactions of the Medical and Chirurgical Faculty of the State of Maryland. Ninety-fifth annual session, held at Baltimore, Md., April, 1893; also semi-annual session, held at Easton, Md., November, 1892, Paper, octavo, pp. 111. Baltimore: Griffin, Curley & Co., Printers, 202 E. Baltimore street. 1893.

These two volumes include the minutes, reports of committees and the president's annual address for the years 1892 and 1893, and the address of the annual orator for 1893, Dr. Reginald H. Fitz, of Boston, who chose for his subject, Intraperitoneal Hemorrhage. The observations of Dr. Fitz are always entertaining and instructive and he was as much so on this occasion as ever. In the course of his paper, Dr. Fitz remarked that "the prevailing idea that intraperitoneal hemorrhage is always a disease of women and is the result of ectopic gestation, has a certain practical value, but is not true. Mild and fatal cases occur in men, though in far less proportions than in women. That the hemorrhage may take place it is essential that blood-vessels rupture. The rupture demands a weakened vascular wall. This weakening is the result of causes which may occur in either sex alike, or may be limited to the female sex." While this statement is an admitted truth, it is none the less true that by far the most frequent cause of intraperitoneal hemorrhage is the rupture of an ectopic gestation sac.

In an explanatory note it is stated that on account of the scarcity of funds, the faculty directed the publication committee to omit the publication of all papers read before the society except the address of the president and the annual oration.

---

Twelfth Annual Report of the State Board of Health of New York. Transmitted to the Legislature, February, 1892. Octavo, pp. 558. Albany: James B. Lyon, State Printer, 1892.

Thirteenth Annual Report of the State Board of Health of New York. Transmitted to the Legislature, March 9, 1893. Octavo, pp. 736, with maps. Albany: James B. Lyon, State Printer. 1893.

Whatever may be said in criticism of the office methods of the State Board of Health, lately under investigation by the legislature, it must be confessed that its official work is well done, as indicated by the volumes before us. We have been at a loss to understand why these reports have appeared at so late a date, but we presume that it is no fault of the board, but rather of the public printer. The work done by the New York State Board of Health will compare favorably with that of other states. These volumes will possess interest to all who are identified with preventive medicine either officially or as a matter of preference.

---

### BOOKS RECEIVED.

A Practical Treatise on Medical Diagnosis, for Students and Physicians. By John H. Musser, M. D., Assistant Professor of Clinical Medicine in the University of Pennsylvania, Philadelphia; President of the Pathological Society of Philadelphia. Octavo, 873 pages, 162 engravings and two colored plates. Cloth, $5.00; leather, $6.00. Philadelphia: Lea Brothers & Co. 1894.

Medical Jurisprudence, Forensic Medicine and Toxicology. By R. A. Witthaus, A. M., M. D., Professor of Chemistry, Physics and Hygiene in the University of the City of New York, etc., and Tracy C. Becker,

A. B., LL. B., Counselor-at-Law and Professor of Criminal Law and Medical Jurisprudence in the University of Buffalo. In four volumes. Volume I. Large 8vo, 845 pages, illustrated with wood-cuts and two lithographic plates in colors. Price, in muslin, $5.00; in brown sheep and in law style, $6.00 per volume. Sold by subscription only. New York: William Wood & Company. 1894.

Tumors, Innocent and Malignant. Their Clinical Features and Appropriate Treatment. By J. Bland Sutton, F. R. C. S., Assistant Surgeon to the Middlesex Hospital, London. In one very handsome octavo volume of 526 pages, with 250 engravings and nine full-page plates. Cloth, $4.50. Philadelphia: Lea Brothers & Co., Publishers. 1894.

The Year-Book of Treatment for 1894. A Comprehensive and Critical Review, for Practitioners of Medicine and Surgery. In a series of twenty-four chapters, by eminent specialists. In one 12mo volume of 497 pages. Cloth, $1.50. Philadelphia: Lea Brothers & Co. 1894.

A Text-book on Diseases of the Eye. By Henry D. Noyes, A. M., M. D. Complete in one octavo volume of 816 pages, profusely illustrated with 269 wood engravings in the text, five chromo-lithographic plates, and ten plates in black and colors. Second revised edition. Price, in cloth, $6.00; leather, $7.00. New York: William Wood & Company. 1894.

An American Text-book of the Diseases of Children, including special chapters on Essential Surgical Subjects; Diseases of the Eye, Ear, Nose and Throat; Diseases of the Skin; and on the Diet, Hygiene and General Management of Children. By American teachers. Edited by Louis Starr, M. D., Physician to the Children's Hospital, and Consulting Pediatrist to the Maternity Hospital, Philadelphia; Late Clinical Professor of Diseases of Children in the Hospital of the University of Pennsylvania; Member of the Association of American Physicians, and of the American Pediatric Society; Fellow of the College of Physicians of Philadelphia, etc. Assisted by Thompson S. Westcott, M. D., Attending Physician to the Dispensary for Diseases of Children, Hospital of the University of Pennsylvania; Physician to Out-Patient Department, Episcopal Hospital; Fellow of the College of Physicians of Philadelphia. Royal 8vo, pp. xiv.—1,190. Illustrated with wood-cuts and twenty-eight half-tone and colored plates. Sold by subscription only. Price, cloth, $7.00; sheep, $8.00; half Russia, $9.00. Philadelphia: W. B. Saunders, 925 Walnut street. 1894.

A Manual of Therapeutics. By A. A. Stevens, M. D., Lecturer on Terminology and Instructor in Physical Diagnosis in the University of Pennsylvania; Demonstrator of Pathology in the Woman's Medical College, Philadelphia, etc., etc. Small 8vo, pp. 435. Price, $2.25. Philadelphia: W. B. Saunders, 925 Walnut street. 1894.

Syllabus of the Obstetrical Lectures in the Medical Department of the University of Pennsylvania. By Richard C. Norris, M. D., Demonstrator of Obstetrics, University of Pennsylvania; Assistant Obstetrician, University Maternity, etc., etc. Third edition. Duodecimo, pp. xviii.—222. Price, $2.00 net. Philadelphia: W. B. Saunders, 925 Walnut street. 1894.

Essentials of Physics. Arranged in the form of questions and answers. Prepared especially for students of medicine. By Fred. J.

Brockway, M. D., Assistant Demonstrator of Anatomy at the College of Physicians and Surgeons, New York. Saunders' Question-Compends, No. 22. Second edition, revised. With 155 illustrations. Duodecimo, pp. 330. Price, $1.00 net. Philadelphia: W. B. Saunders, 925 Walnut street. 1894.

Transactions of the Medical Society of the State of North Carolina. Fortieth annual meeting, held at Raleigh, N. C., May 9, 10 and 11, 1893. Octavo, paper, pp. 154. Wilmington, N. C.: Jackson & Bell. 1893.

A Manual of Minor Surgery and Bandaging, for the Use of House-Surgeons, Dressers and Junior Practitioners. By Christopher Heath, F. R. C. S., Surgeon to University College Hospital and Holme Professor of Clinical Surgery in University College, London; Member of the Council of the Royal College of Surgeons of England. Tenth edition. Illustrated, 16mo, pp. xvi.—389. Price, $2.00. Philadelphia: P. Blakiston, Son & Co., 1012 Walnut street. 1894.

A Text-Book of the Diseases of Women. By Henry J. Garrigues, A. M., M. D., Professor of Obstetrics in the New York Post-graduate Medical School and Hospital; Gynecologist to St. Mark's Hospital in New York City; Gynecologist to the German Dispensary in the City of New York; Consulting Obstetrician to the New York Infant Ayslum; Obstetric Surgeon to the New York Maternity Hospital; Fellow of the American Gynecological Society; Fellow of the New York Academy of Medicine; President of the German Medical Society of the City of New York, etc. Octavo, pp. 690, containing 310 engravings and colored plates. Price, cloth, $4.00 net; sheep, $5.00 net. Philadelphia: W. B. Saunders, 925 Walnut street. 1894.

A Primer of Psychology and Mental Disease. By C. B. Burr, M. D., Medical Superintendent of the Eastern Michigan Asylum; Member of the American Medico-Psychological Association, the American Medical Association, the State Medical Society, the Pontiac Medical Society; Corresponding Member of the Detroit Medical and Library Association. Duodecimo, pp. vi.—104. Price, $1.00. Detroit, Mich.: George S. Davis.

Clinical Lectures on Pediatrics, delivered in the Vanderbilt Clinic during the Session of 1892–93. By A. Jacobi, M. D., Clinical Professor of the Diseases of Children in the College of Physicians and Surgeons of New York, etc., etc. (Stenographic reports.) Reprinted from *Archives of Pediatrics*, Volume X., 1893. Octavo, pp. 195. New York: Baily & Fairchild. 1893.

Manuel de Médecin Practicien, la Pratique Journalière de la Chirurgie, dans Les Hopitaux de Paris, Aide-Mémoire et Formulaire de Thérapeutique Appliquée par le Professeur Paul Lefert. Paris Librairie J.—B. Baillière et Fils. 1894.

